# Supplementary material for: Landscape dynamics and the Phanerozoic diversification of the biosphere
Source: Nature. 2023 Nov 29;624(7990):115–21. doi: 10.1038/s41586-023-06777-z (PMC10700141; doi:10.1038/s41586-023-06777-z)
Supplement: Supplementary file 1 — Supplementary Figs. 1 and 2 and Table 1, and legends for Supplementary Videos 1–4. [file 41586_2023_6777_MOESM1_ESM.pdf]

nature portfolio

https://doi.org/10.1038/s41586-023-06777-z

Supplementary information

Landscape dynamics and the Phanerozoic diversification of the biosphere

In the format provided by the authors and unedited

Supplementary Information for

Landscape dynamics and the Phanerozoic diversification of the biosphere

Tristan Salles[1,*,+], Laurent Husson[2,+], Manon Lorcery[1,2], and Beatriz Hadler Boggiani[1]

[1]School of Geosciences, The University of Sydney, Sydney, Australia
[2]CNRS, ISTerre, Université Grenoble-Alpes, Grenoble, France
[+] T.S. and L.H. contributed equally to this work.

*Corresponding Author: tristan.salles@sydney.edu.au

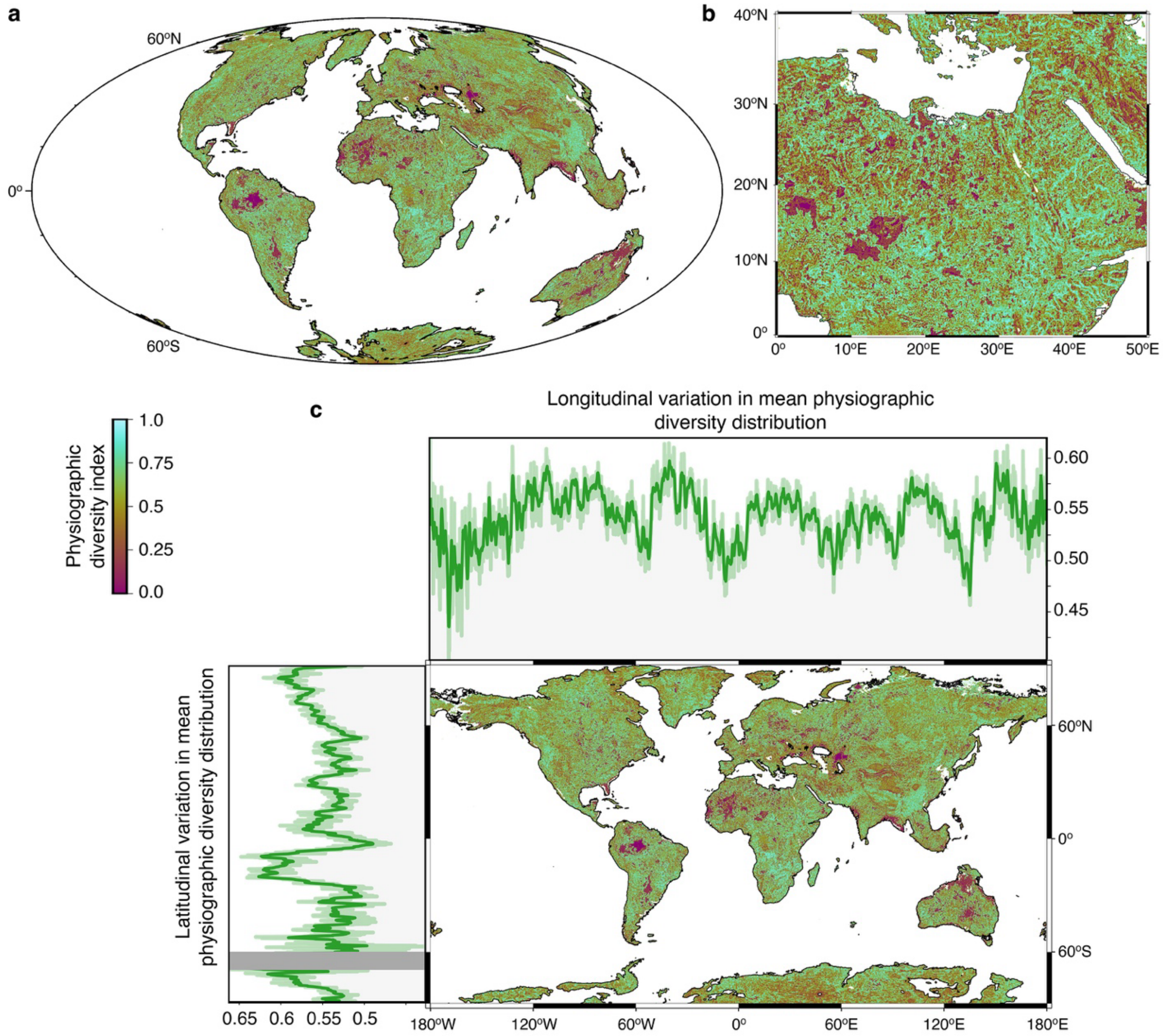

Supplementary Figure 1. Physiographic diversity index. **a.** Shannon index derived from the paleo-physiography morphometrics at global scale and **b.** for Northeast Africa (chosen example at 26 Ma). **c.** Longitudinal and latitudinal distribution of the physiographic diversity index P_{DIV} aggregated over 0.5° bands with light green range representing the 5th to 95th percentile range.

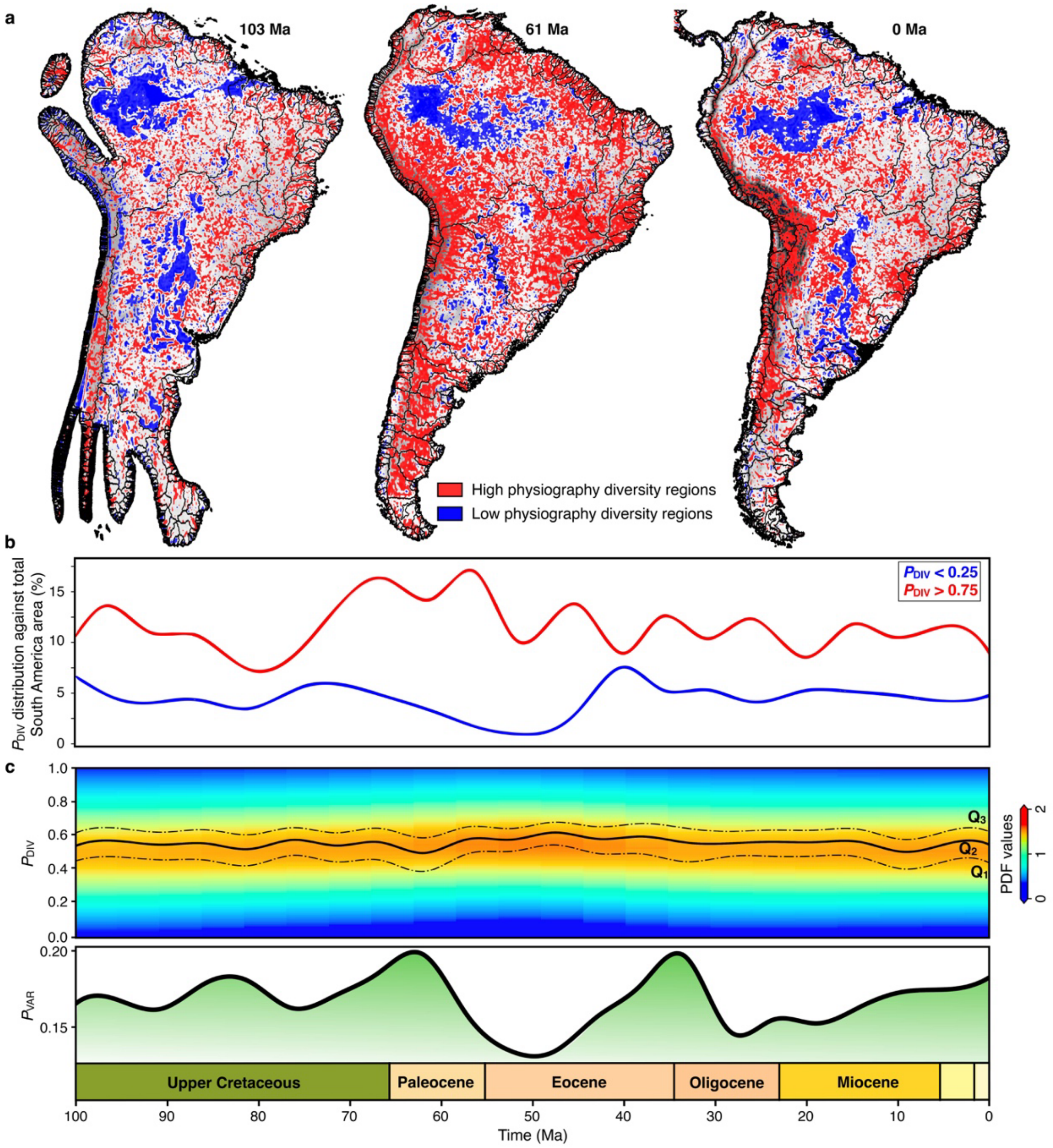

Supplementary Figure 2. Evolution of South America physiographic diversity index since the Upper Cretaceous. **a.** Three maps highlighting different stages of physiographic distributions with (1) limited high physiographic diversity zones during the early phases of the Andean Mountains building in the Upper Cretaceous, (2) a pulse of high diversity regions across the continent following the drying of the interior seaway during the Paleocene, and (3) high physiographic diversity mostly concentrated on the Andes at present-day. **b.** South America average temporal trends in physiographic diversity areas (percent of continental area) for high (red – $P_{DIV} > 0.75$) and low values (blue – $P_{DIV} < 0.25$). **c.** South America temporal change of P_{DIV} (with lower quartile Q_1 (25%), median (Q_2), and upper quartile Q_3 (75%)) and estimated likelihood for P_{DIV} (probability density function – PDF). Bottom panel shows the corresponding physiographic variety index (P_{VAR}).

Supplementary Table 1. Hierarchical classification values. Top table shows the parametrisation for the slope and water flux variables. Bottom table describes the classification of landforms made by combining the two observation scales of the standardized topographic position index (TPI_{SF}|TPI_{SC} for fine and coarse respectively).

	**Category**						
	**1**	**2**	**3**	**4**	**5**	**6**	**7**
**Slope** (degrees)	<0.125	≥0.125	≥0.25	≥0.5	≥0.75	≥1.5	≥3
**Water flux** (Log-scale m^3/yr)	<7	≥7	≥8	≥9	≥10		

Coarse scale TPI_{SC}

**Fine scale TPI_{SF}**			
**>100**	**3**<br>Upland incised drainages Stream headwaters	**7**<br>Flat ridge tops Mesa tops	**10**<br>Mountain tops High narrow ridges
	**2**<br>Midslope incised drainages Local valleys in plains	**5** or **6**<br>**5** – Broad flat areas (slope<0.125)<br>**6** – Broad open slopes (slope≥0.125)	**9**<br>Midslope drainage divides Local ridges in plains
**<-100**	**1**<br>V-shape river valleys Deep canyons	**4**<br>U-shape valleys	**8**<br>Local ridge/hilltops within broad valleys
	**<-100**		**>100**

Supplementary Video 1. Phanerozoic landscape dynamics. Simulated physiographies under surface processes action showing continental topography evolution and river networks (dark blue).

Supplementary Video 2. Phanerozoic erosion and deposition evolution for each time slice. Simulated erosion/deposition rates (blue/red respectively) under riverine and hillslope processes.

Supplementary Video 3. Phanerozoic distribution of sediment flux to the ocean. Positions of river mouths delivering the largest volume of sediments to the ocean (500th largest rivers). Circles color and size are scaled based on sediment flux values.

Supplementary Video 4. Phanerozoic distribution of water flux to the ocean. Positions of river mouths delivering the largest volume of water to the ocean (500th largest rivers). Circles colour and size are scaled based on water flux values.
