# Supplementary figures and images for: Landscape dynamics and the Phanerozoic diversification of the biosphere
Source: Nature. 2023 Nov 29;624(7990):115–21. doi: 10.1038/s41586-023-06777-z (PMC10700141; doi:10.1038/s41586-023-06777-z)

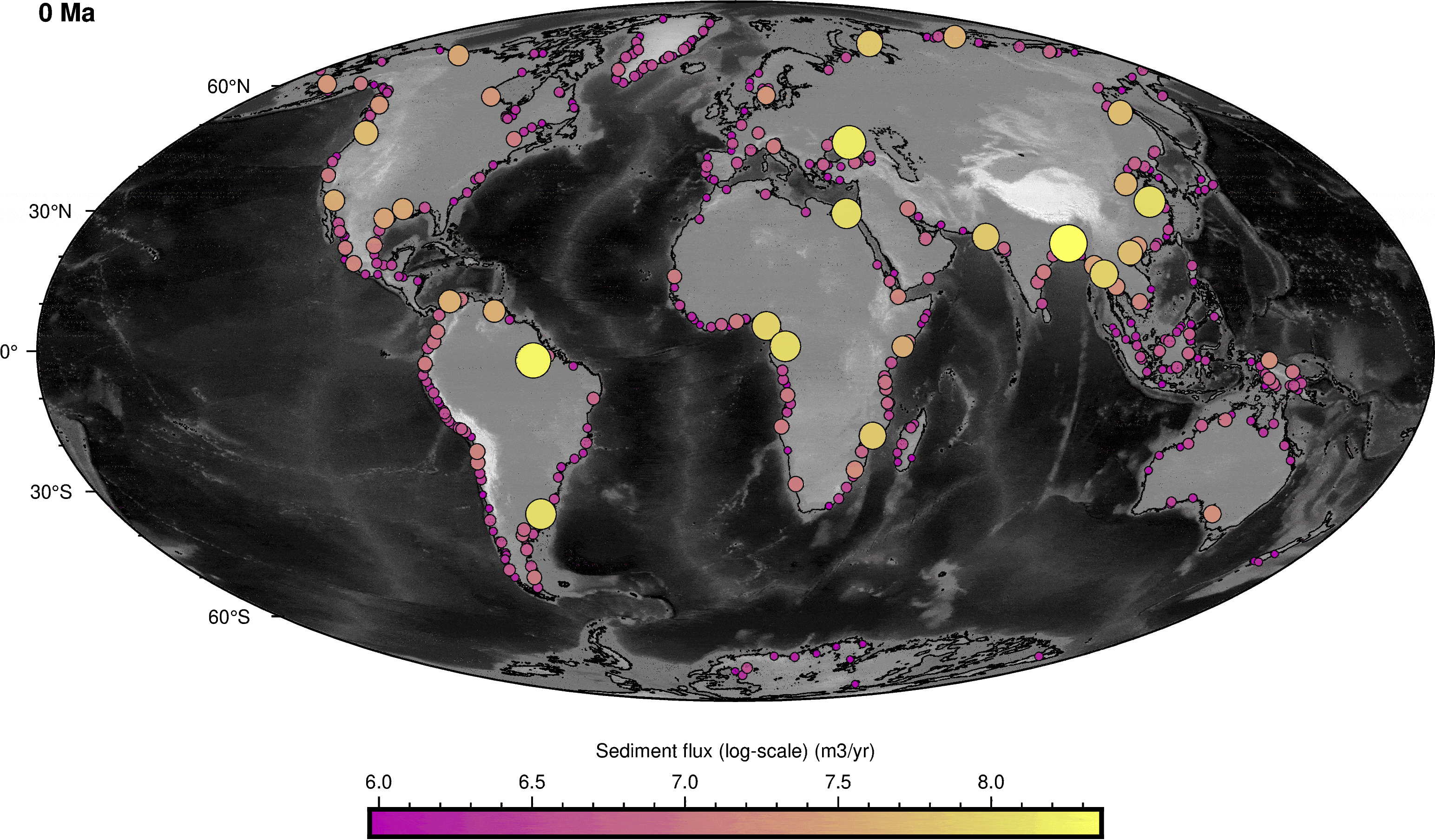

Supplement: Supplementary file 6 — Phanerozoic distribution of sediment flux to the ocean. [file 41586_2023_6777_MOESM6_ESM.gif]

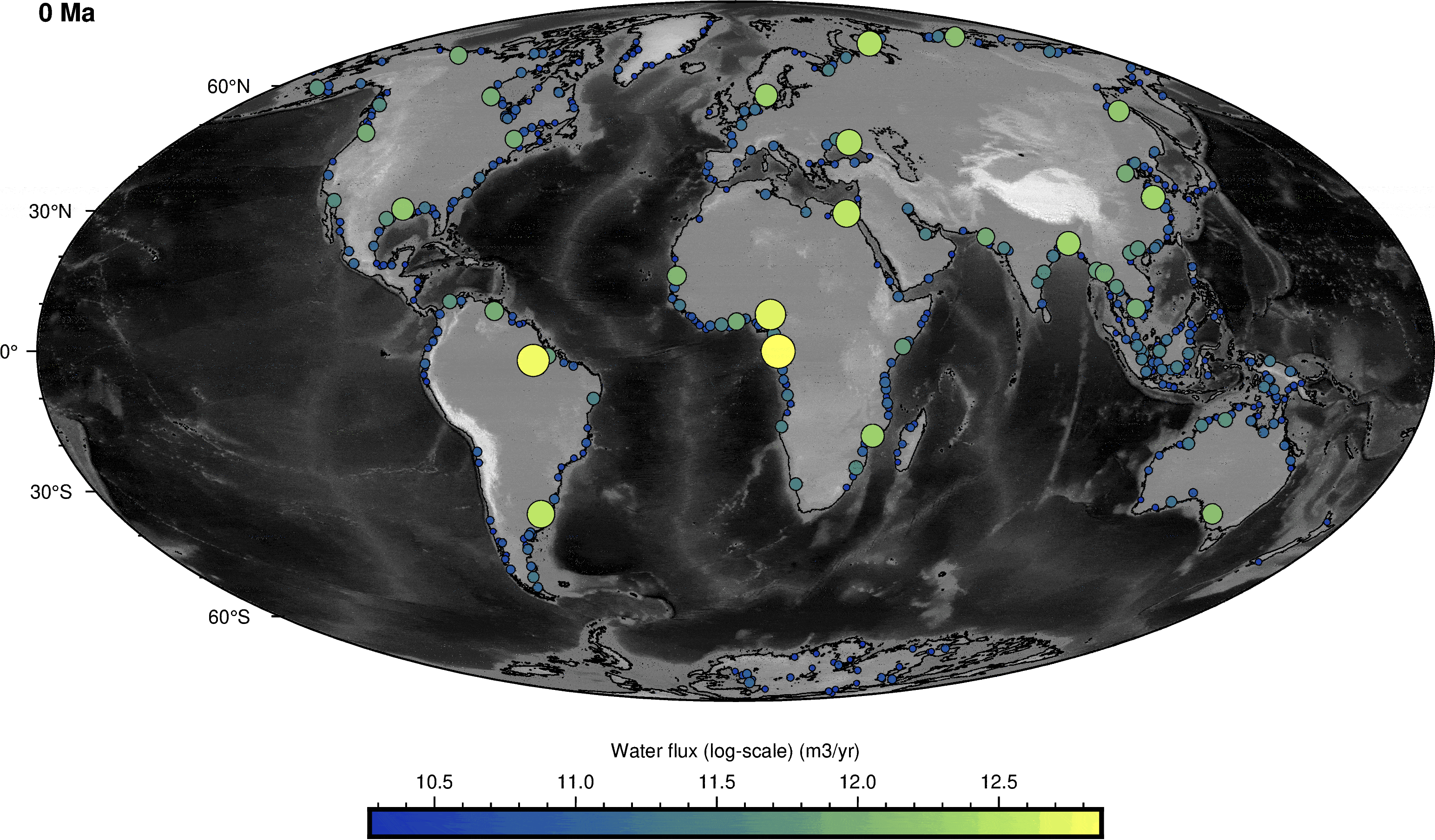

Supplement: Supplementary file 7 — Phanerozoic distribution of water flux to the ocean. [file 41586_2023_6777_MOESM7_ESM.gif]
